# Hypertension and Diagnosis of Parkinson’s Disease: A Meta-Analysis of Cohort Studies

**DOI:** 10.3389/fneur.2018.00162

**Published:** 2018-03-19

**Authors:** Liyan Hou, Qiujuan Li, Liping Jiang, Hongyan Qiu, Chengyan Geng, Jau-Shyong Hong, Huihua Li, Qingshan Wang

**Affiliations:** ^1^School of Public Health, Dalian Medical University, Dalian, China; ^2^Department of Epidemiology and Health Statistics, School of Public Health, Ningxia Medical University, Ningxia, China; ^3^Laboratory of Neurobiology, National Institute of Environmental Health Sciences, National Institutes of Health, Research Triangle Park, NC, United States; ^4^Department of Cardiology, Institute of Cardiovascular Diseases, First Affiliated Hospital of Dalian Medical University, Dalian, China

**Keywords:** hypertension, Parkinson’s disease, cohort studies, meta-analysis, Parkinson’s disease diagnosis

## Abstract

**Background:**

Hypertension has been associated with cognitive dysfunction in the general population and patients with Alzheimer’s disease (AD). However, there are contradictory data regarding the potential association between hypertension and diagnosis of Parkinson’s disease (PD), the second most common neurodegenerative disorder after AD. The purpose of this meta-analysis is to synthesize data from cohort studies to explore the potential association between preexisting hypertension and subsequent PD diagnosis.

**Methods:**

The PubMed and Embase databases were searched to identify all relevant studies. Two independent investigators performed the data extraction. Eligible cohort studies providing risk and precision estimates related to hypertension and PD were selected. Pooled risk ratios (RRs) with 95% confidence interval (CI) were calculated by using a random-effects model or a fixed-effects model. Sensitivity analyses after excluding one study at a time were performed to assess the stability of the results. Publication bias was assessed with Begg’s test and Egger’s test.

**Results:**

Seven cohort studies were identified, including 3,170 persons who were confirmed to have developed PD and 339,517 participants who did not have PD during follow-up. The onset of hypertension before PD diagnosis was significantly associated with an increased risk of motor stage PD (RR = 1.799, 95% CI [1.066–3.037]). This relationship was further confirmed by secondary analyses based on estimates adjusted for potential vascular confounders (RR = 1.319, 95% CI [1.073–1.622]). After excluding one study at a time, the sensitivity analyses still showed that hypertension history was significantly associated with an increased risk of motor stage PD (RR with 95% CI ranging from 1.11 [1.075–1.35] to 1.42 [1.65–1.83]). No publication bias was observed in this meta-analysis.

**Conclusion:**

The findings of this meta-analysis suggest that hypertension may be a risk factor for motor stage PD, which may provide novel insights into the etiology and pathogenesis of this neurodegenerative disorder. However, large-scale well-designed studies that consider various confounders are still needed to further verify and clarify the association between hypertension and PD diagnosis.

## Introduction

The incidence of Parkinson’s disease (PD), which is the second most common neurodegenerative disorder after Alzheimer’s disease (AD), rises rapidly with age and affects more than 1.7% of the population over 65 years of age (1). As populations age globally, the number of individuals with PD worldwide will increase more than double by 2030 (2). Traditionally, PD was considered a motor disorder characterized by the progressive loss of dopaminergic neurons in the substantia nigra pars compacta and the presence of Lewy bodies mainly composed of α-synuclein in remaining neurons (3). PD is now recognized as having a more complex phenotype and widespread α-synuclein pathology that starts in the myenteric plexus and/or the olfactory system and progresses upwardly in a prion-like fashion (4, 5). In addition to classic motor impairments (6), many non-motor symptoms, such as constipation, fatigue, sleep disorder, depression, apathy, and anxiety, are also identified in patients with PD (7, 8), and these symptoms occur even before the onset of motor deficits and greatly decrease the quality of life of patients (9). Current treatments for PD patients provide symptom relief of motor impairments but fail to improve the non-motor symptoms and the progressive neurodegenerative process (6, 10).

The etiology of PD is largely unknown, which greatly hampers the development of novel preventive or therapeutic strategies for combating this neurodegenerative disorder. PD is a multifactorial brain disorder, in which both genetic and environmental factors play important roles. The inherited forms of PD account for only 10–15% of all cases, and the majority of PD cases are likely due to different combinations of environmental exposures and genetic susceptibility (11). However, at present, the environmental risk factors for developing PD are incompletely known. Considering that typical PD neuropathology may be encountered in clinically healthy individuals (12), we assumed that certain environmental factors might be risk factors for the severity of the motor manifestation of PD.

Abnormalities in blood pressure (BP) due to autonomic dysfunction can occur even in the early stages of PD, often preceding the onset of the classic motor symptoms of PD (13). In addition to orthostatic and postprandial hypotension, PD patients also experience nocturnal and supine hypertension, which suggests that BP regulation is impaired in these patients (13, 14). According to Tsukamoto’s report, nocturnal hypertension exists in up to 64.9% of patients with PD (14). Accumulating evidence suggests that hypertension is associated with cognitive dysfunction in the general population and patients with AD (15–22). Since supine hypertension may be a sign of pre-motor PD (23), we hypothesized that preexisting hypertension might result in faster progression of nigral dopaminergic neurodegeneration and related motor symptoms. However, epidemiological studies on the association between hypertension and motor stage PD/PD diagnosis have generated inconclusive results to date, with some studies suggesting an association (24–27) and others not (28–30). To address the inconsistency of the relationship between hypertension and motor stage PD/PD diagnosis, a meta-analysis was conducted. Our study may enhance our understanding for the etiology of motor stage PD and therefore improve clinical practice in term of prevention for patients suffering from this neurodegenerative disorder.

## Materials and Methods

### Literature Search Strategy

Our meta-analysis adhered to the Preferred Reporting Items for Systematic Reviews and Meta-analyses statement (31) and was written according to the Meta-analysis of Observational Studies in Epidemiology guidelines (32).

The PubMed and Embase databases were searched to identify all relevant literature. The following search strategy was used: (“Parkinson’s disease” OR “Parkinson disease”) AND (“hypertension” OR “high blood pressure” OR “high blood pressures”) in Title/abstract. The subjects of studies and the languages of articles were limited to humans and English, respectively. If more than one article was found that used the same data, we included the study with the largest sample size only. We undertook a database search from April 15 to May 1, 2017.

### Inclusion Criteria

Studies meeting the following criteria were included in this meta-analysis: (1) evaluated the association between hypertension and PD risk; (2) had a cohort design; (3) had clearly stated diagnostic criteria for PD and hypertension; (4) the study population had no history of PD; and (5) the study reported at least a risk [relative risk (RR) or hazard ratio (HR)] with 95% confidence interval (CI) or provided sufficient data to calculate them.

### Exclusion Criteria

Studies were excluded if they met the following criteria: (1) cohort studies that lack the systematic case identification or have bias in patient ascertainment (for PD); (2) did not provide RR/HR and RR/HR could not be calculated from the results; (3) studies without original data such as comments, letters, or reviews; and (4) case–control study design. The case–control study design was not included mainly due to the high burden of orthostatic hypertension observed in PD patients (33), which might mask the actual association between hypertension and PD diagnosis. The other reason was the selection bias among the case–control studies. A previous study showed that the use of antihypertensive agents, especially calcium channel blockers, is associated with a reduced incidence of PD in hypertensive patients (34). People with hypertension are more likely to seek health-care service than normotensive people, which may reduce the prevalence of PD in hypertensive populations.

### Data Extraction

Two authors (Liyan Hou and Liping Jiang) independently assessed the studies according to the inclusion/exclusion criteria and any discrepancies were resolved by discussion with senior authors (Qingshan Wang and Huihua Li). The extracted information included the first author, publication year, geographical location, study design, number of participants, sex ratio, follow-up duration, PD and hypertension diagnostic criteria, risk estimates for PD and analytical methods. The Newcastle Ottawa scale was used to access the methodological quality of the studies (35) by two authors independently. The maximum score of 9 stars reflects the highest quality, 7–8 stars reflects medium quality, and ≤6 stars reflects low quality.

### Data Analyses

Meta-analysis of the relationship between hypertension and risk of subsequent motor PD was conducted to calculate the pooled RR and corresponding 95% CI. The heterogeneity across studies was assessed using the Cochran’s *Q* and *I*^2^ statistics (36), and a value of *I*^2^ > 50% was considered to indicate significant heterogeneity (37, 38). In the presence of heterogeneity, pooled RR and corresponding 95% CI were calculated by using a random-effects model (39). Otherwise, the fixed-effects model was used. The significance of pooled RR was determined by a *Z*-test. Meta-regression analysis was used to assess the potentially important covariates that might exert a substantial impact on between-study heterogeneity (40). Sensitivity analyses after excluding one study at a time were performed to assess the stability of the results (40). Publication bias was assessed using Begg’s test and Egger’s test (37). All analyses were performed using the STATA 12 software. Statistical significance was considered to be *P* < 0.05.

## Results

### Literature Search and Characteristics of Selected Studies

**Figure 1:**
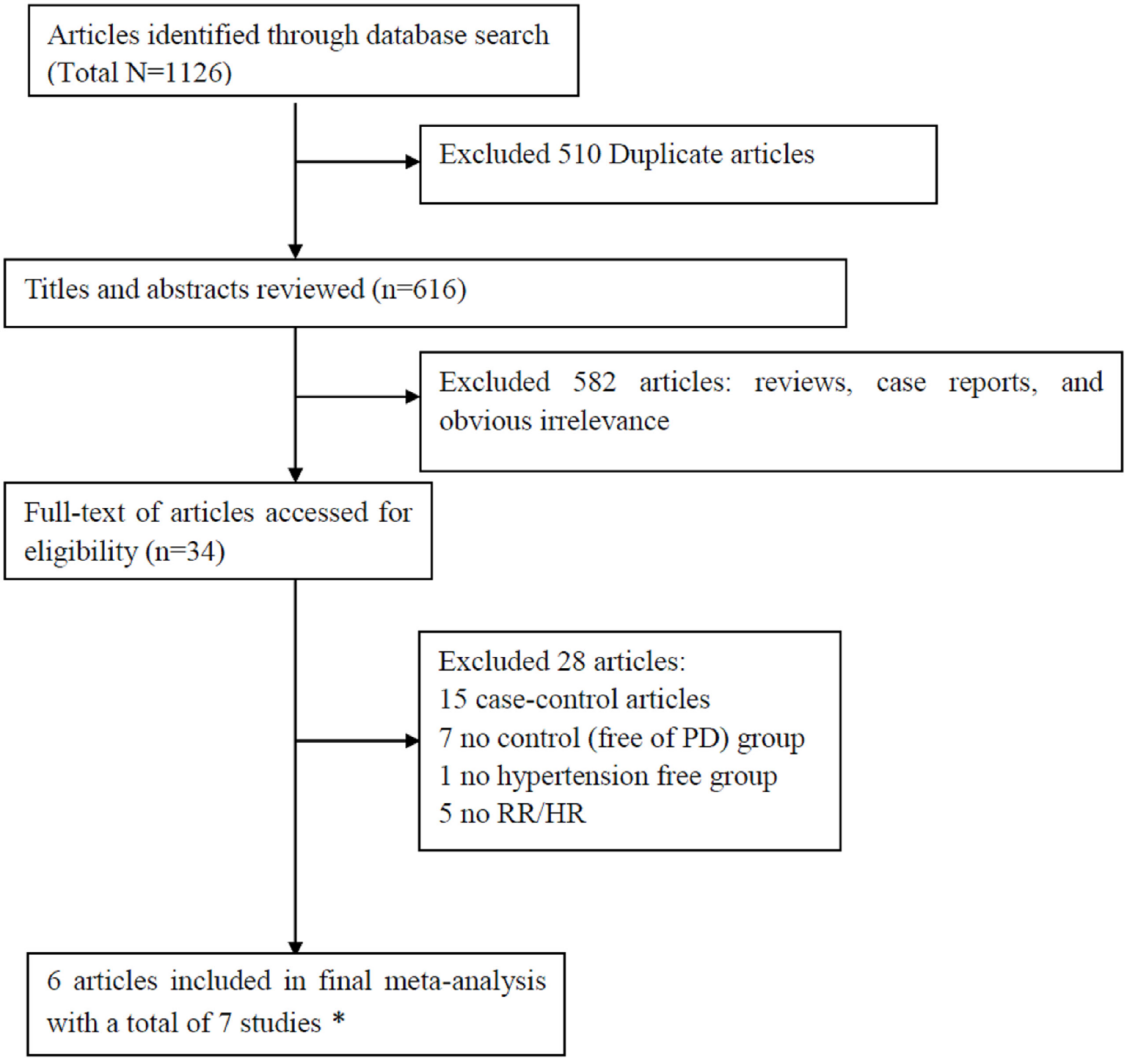
Preferred Reporting Items for Systematic Reviews and Meta-analyses flowchart of the literature search.

The initial search identified a total of 1,126 records in which 510 studies were duplicates. Scanning the titles and abstracts resulted in the exclusion of 582 studies. After detailed assessment of the remaining 34 full-text studies, 28 studies were excluded since they did not provide RR (or HR) with 95% CI (5 studies), lacked a non-PD group (7 studies), lacked a hypertension-free group (1 study), or were case–control designs (15 studies). The excluded studies with the corresponding reasons for exclusion were presented in Figure 1. Since the data in one study were separated by sex, we analyzed the data separately and considered them to be separate studies (24). Therefore, a total of seven studies (24–27, 41, 42) were included in this meta-analysis. Based on NOS, all the studies were assigned a medium-high score (Table 1).

**Table 1:** Characteristics of cohort studies included in the meta-analysis.

Reference, country	Name of cohort	No. of PD/control	Sex (male,%)	Age at baseline (years)	Risk [RR/hazard ratio (HR), 95% confidence interval] for PD	Follow-up duration (starting-ending)	Hypertension diagnostic criteria	PD diagnostic criteria	Statistic used to estimate RR/HR	Star
Saaksjarvi et al. (42), Finland	Mini-Finland Health Survey	86/6,404	46.7	30–79	1.07 (0.55–2.07)*1.13 (0.58–2.20)	30 years (1978–1980 to 2008)	Elevated: systolic blood pressure (SBP) ≥ 130 mmHg or diastolic blood pressure (DBP) ≥85 mmHg or antihypertensive drug treatment. Normal: not elevated	PD cases (ICD-10 code G20) were ascertained through the nationwide registry of the Social Insurance Institution of patients receiving medication reimbursement	Cox proportional hazards model	8

Lai et al. (25), Taiwan	Taiwan National Health Insurance Program	252/24,628	61.01	≥65	2.16 (1.61–2.89)*1.7 (1.26–2.3)	1–10 years (2000–2010 to 2010)	Records. ICD-9, data were recorded for three or more visits in the ambulatory care unit	ICD-9 codes, PD case were ascertained through a health insurance database	Multivariable Cox proportional hazards regression	8

Lai et al. (26), Taiwan	Taiwan National Health Insurance Program	1,268/50,700	45.4	40–84	2.06 (1.83–2.30)*1.42 (1.26–1.61)	9–11 years (1999–2002 to 2011)	Records, ICD-9	ICD-10 codes, PD case were ascertained through a health insurance database	Multivariable Cox proportional hazards regression	8

Lin et al. (27), Taiwan	Taiwan National Health Insurance Program	396/41,469	46.8	≥20	6.52 (5.31–8.01)*1.72 (1.33–2.24)	1–10 years (2000–2011 to 2010)	Records, ICD-9-CM codes 401–405	ICD-9-CM code 332	Multivariable Cox proportional hazards regression	8

Simon et al. (41), United States	Nurses’ Health Study	530/171,349	29.6	Women 30–55Men 40–75	0.96 (0.81–1.15)	Women: 22.9 years (1976–1998); men 12.6 years (1986–1998)	Hypertension—a self-report of doctor-diagnosed hypertension, SBP > 160 mmHg, DBP > 90 mmHg, or reported use of antihypertensive medication	The treating neurologists to complete a questionnaire to confirm the diagnoses of PD and the certainty of the diagnosis (definite, probable, possible) or to send a copy of the medical records	Cox proportional hazards model	8

Qiu et al. (24), Finland	National FINRISK Study	340/21,613	100	45 ± 11.8	1.06 (0.75–1.50)*0.90 (0.63–1.28)	18.8 years (1972–2006)	Blood pressure (BP) was measured at the study site by specially trained nurses. Normal BP (<130/80 mm Hg), high-normal BP (130–139/80–89 mm Hg), hypertension (≥140/90 mm Hg or use of antihypertensive drugs)	The diagnosis of PD has to be based on medical history and the presence of primary symptoms and signs (tremor, bradykinesia, rigidity, and postural instability) determined by comprehensive clinical assessments. Furthermore, the diagnosis has to be done by a specialist in Neurology	Cox proportional hazards model	9

Qiu et al. (24), Finland	National FINRISK Study	298/23,354	0	44.9 ± 11.6	1.73 (1.17–2.56)*1.62 (1.09–2.42)	18.8 years (1972–2006)	BP was measured at the study site by specially trained nursesNormal BP (<130/80 mm Hg), high-normal BP (130–139/80–89 mm Hg), hypertension (≥140/90 mm Hg or use of antihypertensive drugs)	The diagnosis of PD has to be based on medical history and the presence of primary symptoms and signs (tremor, bradykinesia, rigidity, and postural instability) determined by comprehensive clinical assessments. Furthermore, the diagnosis has to be done by a specialist in Neurology	Cox proportional hazards model	9

*PD, Parkinson’s diseases; ICD-9, International Classification of Disease, 9th revision; ICD-10 codes, International Classification of Disease, 10th revision*.

*RR/HR after adjustment for potential vascular confounder.

### Hypertension and Risk of Subsequent Motor PD

Seven cohort studies published between 2007 and 2015 included 3,170 persons who met the criteria for PD diagnosis and 339,517 participants who had not met the criteria for PD diagnosis during follow-up. Among them, four studies reported that hypertension is associated with an increased risk of PD (24–27), whereas the other three studies indicated that there was no significant association (24, 41, 42).

**Figure 2:**
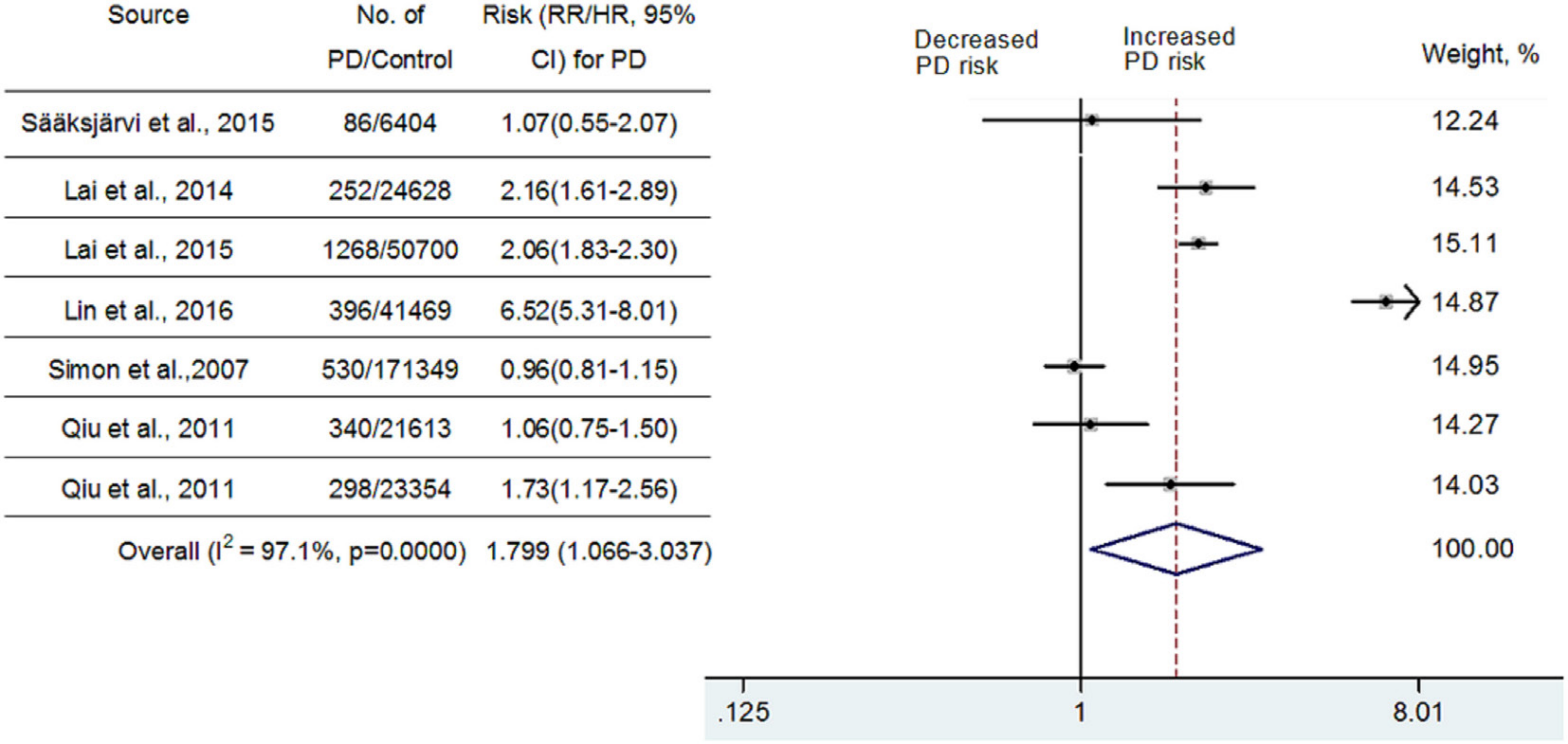
Meta-analysis of the association between hypertension and Parkinson’s disease (PD) risk.

**Figure 3:**
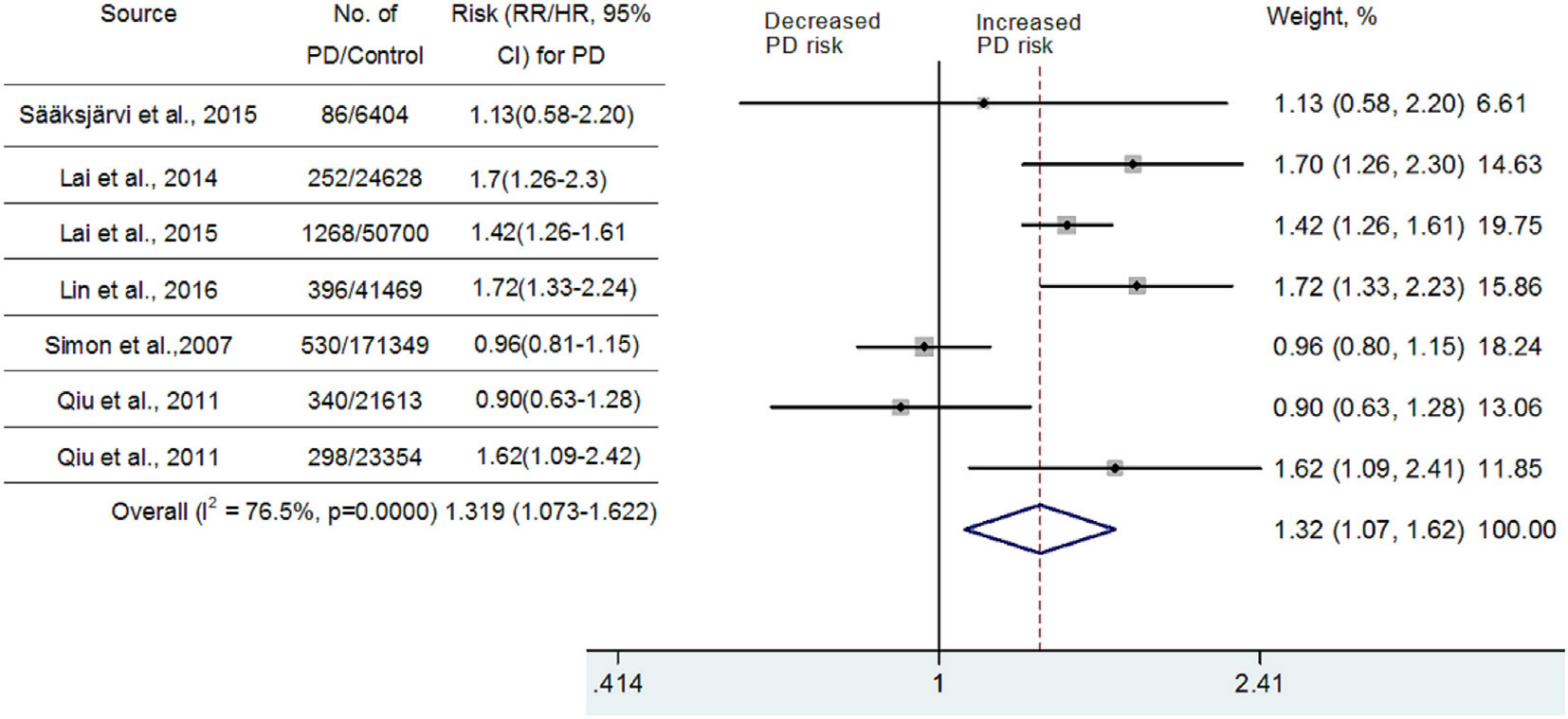
Meta-analysis of the association between hypertension and Parkinson’s disease (PD) risk after adjustment for the potential confounding vascular factors.

Because significant heterogeneity (heterogeneity *P* = 0.0000, *I*^2^ = 97.1%,) was observed, a random-effects model was chosen to calculate pooled RR and corresponding 95% CI. Pooled analysis based on adjusted estimates of seven cohort studies showed that hypertension history before the onset of PD was significantly associated with an increased risk of PD (RR = 1.799, 95% CI [1.066–3.037], Figure 2). Although three cohort studies that were included (25–27) came from Taiwan National Health Insurance, we verified with the author that these three studies were performed separately and contained a very limited number of overlapping individuals. Similarly, we also verified with the authors that two studies performed in Finland (24, 42) contain a very limited number of overlapped individuals. Since diabetes, hyperlipidemia, cerebrovascular disease, and coronary artery disease may contribute to vascular parkinsonism (43), a secondary analysis was carried out after adjustment for potential vascular confounders. As seen in Figure 3, the association remained significant (RR = 1.319, 95% CI [1.073–1.622]; *P* = 0.0000, *I*^2^ = 76.5%). In one study, researchers did not investigate whether vascular disorders could influence the combined estimates or not. The exclusion of this study still showed a significant association between hypertension and PD (RR = 1.429, 95% CI [1.199–1.704]; *P* = 0.0686, *I*^2^ = 52.8%, Figure 3). We conducted a subgroup analysis by ethnicity as well (Asian vs Caucasian). The results showed that hypertension was a risk factor for PD in an Asian population (RR = 1.523, 95% CI [1.338–1.735]) but not in a Caucasian population (RR = 1.083, 95% CI [0.84–1.396]).

Substantial heterogeneity was noted in this meta-analysis. However, as seen in Figure 2, almost all of the seven included cohort studies showed a similar effect, although three of them showed no significant association, which suggested that the partial heterogeneity might come from the variations in the magnitude of the estimated risk instead of the direction. Seven studies that adjusted risk factors for confounders were mostly adjusted for different confounders. The results indicated that adjustment for different confounders was the main source of heterogeneity. In addition, the impact of heterogeneity (*I*^2^) was reduced 20.6% after adjustment for the possibility of confounding vascular factors. After exclusion of one study that did not investigate the effects of vascular disorders on the combined estimates, the impact of heterogeneity was further reduced 23.7%. Since the *I*^2^ (52.8%) was still slightly higher than 50%, meta-regression analysis was further used to explore the other sources of heterogeneity. However, all the variables, including publication year, number of PD diagnoses during follow-up, follow-up duration, and sex ratio, could not explain the source of heterogeneity.

### Sensitivity Analyses and Publication Bias Evaluation

The sensitivity analyses performed by excluding one study at a time revealed that hypertension history before the diagnosis of PD was significantly associated with increased risk of PD. The values of RRs with 95% CIs ranged from 1.11 [1.075–1.35] to 1.42 [1.65–1.83].

**Figure 4:**
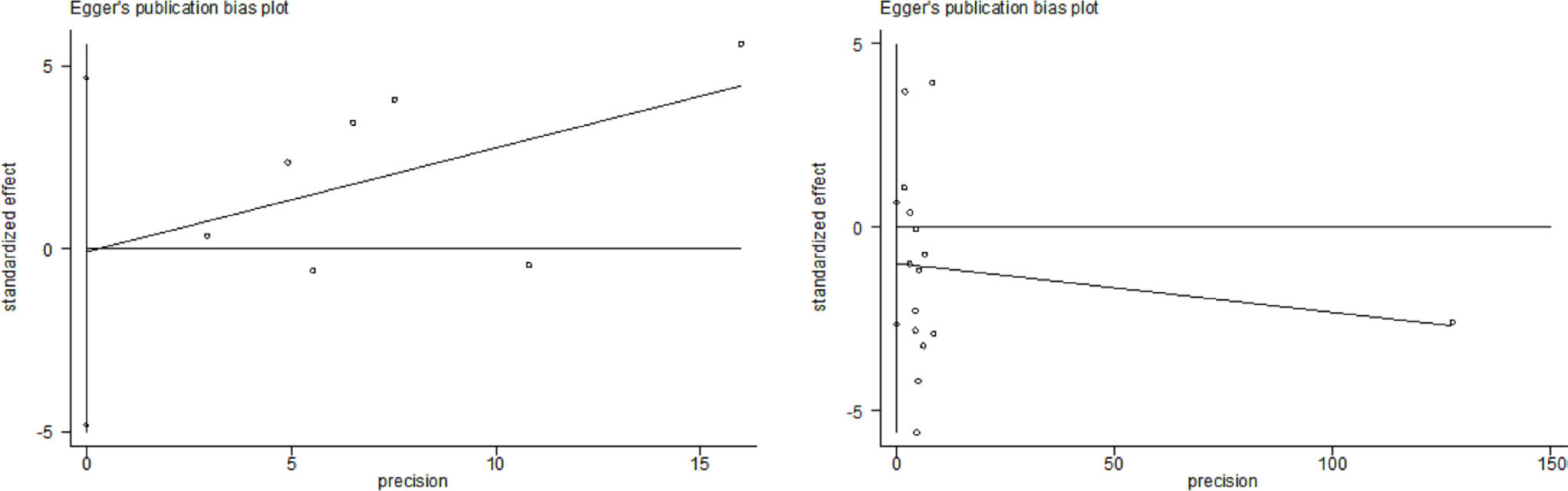
Begg’s test and Egger’s test in cohort studies.

All studies included in this meta-analysis were considered high quality (Table 1). Publication bias was not detected in these studies (*P* = 0.548 and 0.790 in Begg’s test and Egger’s test, respectively) (Figure 4).

## Discussion

To the best of our knowledge, this meta-analysis is the first that aimed to quantitatively investigate the association between hypertension and motor stage PD/PD diagnosis. Here, we found that preexisting hypertension may be a risk factor for PD diagnosis. Our conclusion should be more credible than that of individual studies since our study involved a total of 3,170 persons who met the criteria for PD diagnosis and 339,517 participants who had not met the criteria for PD diagnosis. Our conclusion was drawn based on the evidence derived from cohort studies. A major advantage of the cohort study is that the study participants completed the hypertension history survey before onset of PD. By contrast, the case–control study mainly depends on recall of the subjects (risk factors) and a real causal relationship between factors is difficult to infer. Therefore, the prospective cohort study design is usually preferred in etiologic epidemiology, although it is more expensive and time-consuming than case–control study design.

The subgroup analyses by ethnicity in this meta-analysis showed that hypertension was significantly associated with increased risk of PD diagnosis in the Asian population, but not in the Caucasian population. It is well documented that the prevalence of both PD and hypertension varies by ethnicity. Zhang and Roman (44) found that the prevalence of PD is lower in China, Japan, and Africa and higher among western industrialized nations, especially United States and Europe. Analysis of different populations living in Northern California also showed that the prevalence of PD is lower in Asians than Caucasians (45). In agreement with PD, a similar low prevalence of hypertension is also observed in Asians compared with Caucasians (46). The genetic differences along with differences in environmental factors exposed between Asians and Caucasians may have, at least partially, accounted for the different frequency of hypertension and PD between ethnicities (47, 48). However, the cause for the ethnic difference on association between hypertension and motor stage PD remains unclear. We suspected that some unknown confounding factors included in this meta-analysis might mask the relationship between hypertension and PD diagnosis in Caucasians. In addition, a possibly insufficient number of studies available for pooling might also be responsible for the ethnic difference on association between hypertension and PD diagnosis. Further clinical and large cohort studies are warranted in the future to confirm this ethnic difference.

Currently, the mechanisms for linking hypertension and motor stage PD have yet to be investigated. Since the pathogenesis of the selective dopaminergic neurodegeneration in the SN is obscure (49), the biological mechanisms linking hypertension to PD can only be speculated on. First, patients with hypertension display decreased resting cerebral blood flow, which may reduce the delivery of oxygen to ischemia-sensitive brain regions such as the SN, which results in dopaminergic neurodegeneration and subsequent motor deficits. Second, chronic high BP causes hypertensive neurovascular unit dysfunction in multiple brain regions, including the basal ganglia, which may result in dopaminergic neurodegeneration in the SN and then reduction of dopamine transmitters in the striatum (24, 50). Third, inflammation is a common mechanism that leads to both hypertension and PD. The inflammatory response that exists in hypertensive patients may contribute to neuroinflammation and related damage of dopaminergic neurons in the SN, leading to motor deficits.

Hypertension contributes to cognitive decline in both the general population and patients with AD. Previous studies suggested that increased BP is associated with increased brain atrophy, gray matter atrophy, and white matter injury (51, 52). Recently, a community-based cohort study showed that midlife systolic hypertension and persistence of systolic hypertension into late life are associated with an elevated risk of incident dementia (53). Similarly, Moonga et al. reported that hypertension is also associated with worse cognitive function in patients with AD (22). Similar to these reports, hypertension was recognized as a risk factor for motor stage PD in our study. The results of this meta-analysis underline once more the importance of early diagnosis and treatment of hypertension, which may be especially critical for those with preclinical or prodromal PD. Considering the results of the meta-analysis, monitoring supine hypertension should be considered, at least in those at risk for PD or fulfilling the 2015 Movement Disorders Society criteria for prodromal PD.

### Limitations and Future Perspectives

Several potential limitations should be addressed for this meta-analysis. First, there were a limited number of eligible studies to obtain raw data regarding hypertension duration, levels of BP, and gender of PD patients for comparison. Our meta-analysis therefore could not provide detailed results on these issues. Second, substantial heterogeneity was observed in the meta-analysis. Although subgroup analysis revealed ethnicity as a potential reason for the heterogeneity, the interpretation of this finding should be cautious due to the small number of studies available for pooling. Meta-regression analysis for geographic locations, publication year, and number of PD individuals failed to reveal the source of heterogeneity, which suggests that other unknown confounders might be involved. Among the subjects, the use of antihypertensive drugs might be one of the important confounders. Nationwide Cohort Studies performed in Finland (24) and Taiwan (34) showed that the usage of antihypertensive drugs is associated with a reduced incidence of PD in hypertensive patients. Due to the limited studies included in this meta-analysis that evaluate the effects of antihypertensive drugs, the confounder of usage of antihypertensive drugs cannot be adjusted. Third, although the diagnostic criteria for PD among studies were consistent, the criteria for hypertension were not. The Nurses’ Health Study performed by Simon et al. (41) used a significantly different definition for hypertension (i.e., more than 160 mmHg instead of equal to or more than 130 mmHg in other studies). Fourth, we included studies published only in English. Eligible articles with other languages were not included in this meta-analysis. Finally, the cohort studies included in this meta-analysis were only performed in Asia, Finland, and United States and our findings might not be extended to populations of other ethnicities.

Further well-designed studies with consideration of different levels of hypertension are still required to verify and clarify the association between hypertension and motor stage PD/PD diagnosis. In addition, the possible relation between ethnicity, PD diagnosis, and hypertension may warrant further investigation due to the different conclusions derived from the subgroup analysis by ethnicity. Furthermore, experimental studies are also needed to explore the biological mechanisms linking hypertensive status to PD.

## Conclusion

The results of the meta-analysis confirm and expand the already available evidence suggesting that hypertension may be a risk factor for motor stage PD/PD diagnosis, especially in Asian populations. This may provide novel insights into the etiology of this neurodegenerative disorder. However, more population-based prospective studies which consider various confounders (e.g., age, sex, hypertension duration, the use of antihypertensive drugs, other cardiovascular risk factors, caffeine, and alcohol intake) are still needed to clarify the association between hypertension and PD diagnosis. The results of the meta-analysis underline once more the importance of early diagnosis and correct treatment of hypertension, which may be especially challenging in those with preclinical and prodromal PD.

## Author Contributions

LH and QW conceived the study and wrote the paper; QL and LJ performed the literature search and data extraction; HQ and CG analyzed and interpreted data; HL and J-SH revised the manuscript.

## Conflict of Interest Statement

The authors declare that the research was conducted in the absence of any commercial or financial relationships that could be construed as a potential conflict of interest.
